# Longitudinal changes in cardiac function in Duchenne muscular dystrophy population as measured by magnetic resonance imaging

**DOI:** 10.1186/s12872-022-02688-5

**Published:** 2022-06-09

**Authors:** Abhinandan Batra, Alison M. Barnard, Donovan J. Lott, Rebecca J. Willcocks, Sean C. Forbes, Saptarshi Chakraborty, Michael J. Daniels, Jannik Arbogast, William Triplett, Erik K. Henricson, Jonathan G. Dayan, Carsten Schmalfuss, Lee Sweeney, Barry J. Byrne, Craig M. McDonald, Krista Vandenborne, Glenn A. Walter

**Affiliations:** 1 Department of Physical Therapy, University of Florida, Gainesville, FL 32610 USA; 2 Department of Statistics, University of Florida, Gainesville, FL 32610 USA; 3 Department of Physiology and Functional Genomics, University of Florida, 1600 SW Archer RD, M552, P.O. Box 1002754, Gainesville, FL 32610 USA; 4 Department of Physical Medicine and Rehabilitation, University of California, Davis, Sacramento, CA 95817 USA; 5 Department of Pediatrics, UC Davis, Sacramento, CA 85817 USA; 6 Department of Medicine, Cardiology, University of Florida, Gainesville, FL 32610 USA; 7 Department of Pharmacology and Therapeutics, University of Florida, Gainesville, FL 32610 USA; 8 Department of Pediatrics, University of Florida, Gainesville, FL 32610 USA

**Keywords:** Cardiac magnetic resonance imaging, Duchenne muscular dystrophy, Cardiac circumferential strain

## Abstract

**Background:**

The lack of dystrophin in cardiomyocytes in Duchenne muscular dystrophy (DMD) is associated with progressive decline in cardiac function eventually leading to death by 20–40 years of age. The aim of this prospective study was to determine rate of progressive decline in left ventricular (LV) function in Duchenne muscular dystrophy (DMD) over 5 years.

**Methods:**

Short axis cine and grid tagged images of the LV were acquired in individuals with DMD (n = 59; age = 5.3–18.0 years) yearly, and healthy controls at baseline (n = 16, age = 6.0–18.3 years) on a 3 T MRI scanner. Grid-tagged images were analyzed for composite circumferential strain (*ℇcc*%) and *ℇcc*% in six mid LV segments. Cine images were analyzed for left ventricular ejection fraction (LVEF), LV mass (LVM), end-diastolic volume (EDV), end-systolic volume (ESV), LV atrioventricular plane displacement (LVAPD), and circumferential uniformity ratio estimate (CURE). LVM, EDV, and ESV were normalized to body surface area for a normalized index of LVM (LVMI), EDV (EDVI) and ESV (ESVI).

**Results:**

At baseline, LV *ℇcc*% was significantly worse in DMD compared to controls and five of the six mid LV segments demonstrated abnormal strain in DMD. Longitudinal measurements revealed that *ℇcc*% consistently declined in individuals with DMD with the inferior segments being more affected. LVEF progressively declined between 3 to 5 years post baseline visit. In a multivariate analysis, the use of cardioprotective drugs trended towards positively impacting cardiac measures while loss of ambulation and baseline age were associated with negative impact. Eight out of 17 cardiac parameters reached a minimal clinically important difference with a threshold of 1/3 standard deviation.

**Conclusion:**

The study shows a worsening of circumferential strain in dystrophic myocardium. The findings emphasize the significance of early and longitudinal assessment of cardiac function in DMD and identify early biomarkers of cardiac dysfunction to help design clinical trials to mitigate cardiac pathology. This study provides valuable non-invasive and non-contrast based natural history data of cardiac changes which can be used to design clinical trials or interpret the results of current trials aimed at mitigating the effects of decreased cardiac function in DMD.

**Supplementary Information:**

The online version contains supplementary material available at 10.1186/s12872-022-02688-5.

## Background

Mutations in the dystrophin gene lead to complete absence or partial production of dystrophin protein, giving rise to Duchenne (DMD) and Becker (BMD) muscular dystrophy, respectively. Respiratory and cardiac failure are the leading causes of demise in the DMD patient population [1–3], and the number of patients presenting with end-stage heart failure has increased significantly over the last decade due to advancements in respiratory care [2–6].

Based on clinical observation, cardiac involvement in DMD is usually asymptomatic at early ages, and clinically appreciative manifestations present around the age of 10 years with ECG abnormalities and sinus tachycardia [2, 5, 7]. As the disease progresses, individuals develop symptoms of diastolic dysfunction which eventually gives way to systolic dysfunction and dilation of the chambers, thinning of the heart walls, and myocardial fibrosis [5, 7]. Myocardial damage at a cellular level precedes clinically significant cardiac dysfunction in DMD [2, 8, 9].

At present, there is no therapy available to reverse the course of cardiomyopathy in DMD, but various cell and viral vector-based therapies targeted towards cardiomyopathy in DMD are in clinical and preclinical trials [10–13]. While promising therapies are in the pipeline, there is still a scarcity of natural history data related to development and progression of cardiomyopathy in DMD in the presence of contemporary preventive care standards [14–16].

Traditionally, transthoracic echocardiography (TTE), ECG, and MRI have all been used for cardiac assessment in this patient population [17, 18]. Though these non-invasive modalities have identified the effect of disease on global volumetric cardiac function, they each have inherent limitations. TTE is sensitive to changes in cardiac function at a younger age, but with an increase in disease severity, there is a decrease in the acoustic window due to fat deposition and development of scoliosis, limiting its sensitivity [3, 19]. On the other hand, MRI with late gadolinium enhancement (LGE) is widely used to detect focal fibrosis in myocardial tissue but is unreliable in identifying diffuse myocardial fibrosis [4, 20, 21]. In addition, the gadolinium from the contrast agent has been reported to accumulate in organs other than the heart after repeated exposure, leading to potential toxicity [22–24]. Based on these limitations, there is a need to develop cardiac assessment tools that are non-invasive, safe, and sensitive to detect cardiac dysfunction at an early stage [2, 3, 19, 25, 26]. Various non-invasive techniques such as circumferential strain using tagged and feature tracking techniques, T1 mapping, and change in volumetric functions have been used to quantify disease progression in DMD [26–29].

Cardiac magnetic resonance imaging (CMR) using a myocardium tagging approach to calculate peak strain has been shown to be a sensitive measure to detect abnormalities in cardiac function and contractility in DMD. Peak strain can be defined as a measure of biomechanical distortion in tissue length with respect to resting length in the heart wall as it contracts [30, 31]. Studies in DMD have reported circumferential peak strain (*ℇcc*%) at the mid-ventricular level is a sensitive marker to examine cardiac involvement and the effect of therapeutic interventions on cardiac muscle [3, 27, 28, 32–34]. A major advantage of *ℇcc*% is its ability to detect changes at both global and regional levels of myocardium without use of any contrast [3, 28, 32].

The aim of this study was to evaluate the amount of change in left ventricular (LV) cardiac function and structure over a period of five years in ambulatory and non-ambulatory boys and young men with DMD without the use of a contrast agents. Additionally, the study investigated the value of measuring *ℇcc*% cross sectionally and longitudinally, in comparison to more traditional LV functional CMR outcomes. We hypothesized that *ℇcc*%, would be a more sensitive longitudinal marker of myocardial remodeling in people with DMD at both the global and segmental level than the more traditionally used LV functional measures. A secondary aim of this study was to determine if change in cardiac functional parameters in DMD is related to loss of ambulation, age, or the use of cardioprotective drugs.

## Methods

Individuals with a confirmed diagnosis of DMD (based on the genetic report and/or muscle biopsy) and unaffected controls were recruited for participation in a prospective longitudinal cardiac MRI study at the University of Florida (UF) and a retrospective study (pretreatment baseline as a part of interventional clinical trial (NCT02964377)) at the University of California Davis (UCD). At UF. participants underwent a 30–45 min cardiac MRI exam at baseline on a Phllips 3T system, and they returned annually for follow-up cardiac MRI exams for up to 9 years. A separate set of retrospective data was used from data acquired at the UCD, and this cohort underwent a similar cardiac MRI protocol as participants at UF at baseline on a Siemens 3 T system. There were no follow-up visits for this cohort. Participants were defined as non-ambulatory if they were unable to complete the 10 m walk/run test independently within 45 s. The institutional review boards at the University of Florida and the University of California Davis approved the respective studies. Prior to participation, parents of each participant (if participant was younger than 18 years) provided written informed consent, and participants themselves gave written assent. For older participants informed consent was obtained from participants themselves.

### MR acquisition

All cardiac MR images at UF were acquired using a 3.0 T whole-body scanner (Philips Achieva Quasar Dual 3T, Philips, Amsterdam, the Netherlands) with a 32-channel torso anterior–posterior cardiac coil. MR images were acquired using a segmented steady-state free precession (SSFP) technique with ECG gating and free breathing. Cinegraphic (cine) long axis two-chamber and four-chamber MR images were acquired from survey scans. Short-axis cine MR images were acquired from the four-chamber view for the left ventricle from base to apex (FOV = 250 × 250 × 91 mm^3^, TR/TE = 3.6/1.8 ms, slice thickness = 7 mm, minimum number of slices = 11, phases/slice = 25–40). An ECG-triggered fast field echo MR imaging sequence was used to acquire myocardial short-axis tagged images (tag spacing = 6–8 mm, FOV = 260 × 260 mm^2^, slice thickness = 7 mm, TR/TE = 5.5/3.3 ms, phases/segment = 14–18) at the left mid-ventricular level. The length of the ventricle (tip of the apex to mitral valve) was bisected to identify the mid-ventricular region (typically at the level of papillary muscles).

All cardiac MR images at UC Davis were acquired on a 3 Tesla Siemens scanner (TIM Trio, Siemens Healthineers, Erlangen, Germany). Cardiac functional imaging was performed using retrospective ECG gating with a segmented SSFP technique after localized shimming and/or frequency adjusting. Subjects performed breath holds after expiration. Following localization of two and four-chamber views, short-axis and four-chamber cine SSFP images were acquired for the left ventricle from cardiac base to apex (FOV = 256 × 192 mm^2^, slice thickness = 6–8 mm, gap = 20%, AVE = 1, TR/TE = 38/1.4 ms). A minimum of 12 slices were acquired with 30 phases/slice. ECG-triggered short axis grid tagged MR images were acquired at the mid-ventricle level. Grid tag spacing was 8 mm. The scan parameters used were: FOV = 192 × 154 mm^2^, slice thickness = 8 mm, flip angle = 10°, TR/TE = 61/2.4 ms, views per segment = 12.

### MRI analysis

All image analyses were performed at UF by two analyzers using FDA-approved software packages to measure cardiac mass and function (Segment;Medviso; Sweden version 2.0 R5585) and Eulerian circumferential strain (Harmonic Phase; Virtue; version 5.4, Myocardial Solutions, Morrisville, NC). There was strong inter and intra-rater reliability for calculation of both strain (inter [r = 0.92], intra [r = 0.96]) and volumetric analysis (inter [r = 0.83], intra [r = 0.94]). Previous studies by Hor et al. [3] and Heiberg et al. [35] have shown the validation and reliability of both these software packages in calculating strain and volumetric functions, respectively. Of all the scans, one tagged image scan, six short-axis scans and eleven four-chambers could not be used because of poor image quality. The number of subjects reported in the tables represents the final subject numbers used for the analysis. Using the Segment software, the short-axis images with the greatest dilation and contraction of the LV cavity were defined as end diastole and systole, respectively. The endocardium and epicardium throughout diastole and systole were automatically segmented for all slices in which myocardium was visible during the cardiac cycle. Following automatic segmentation, myocardium outlines were manually adjusted to accurately delineate the endo and epicardium prior to final measure calculations. Measures obtained from this analysis include left ventricular mass (LVM), left ventricle ejection fraction (LVEF), left ventricle end systolic volume (LVESV), and left ventricle end diastolic volume (LVEDV). To account for short stature in DMD, volumetric functions and masses were normalized to body surface area (BSA) to calculate left ventricular mass index (LVMI), left ventricular end systolic volume index (LVESVI), and left ventricular end diastolic volume index (LVEDVI). BSA was calculated as described previously described [36, 37]. In a separate analysis, left ventricle atrioventricular plane displacement (LVAPD) was derived from the long axis two chamber and four chamber images (averaged together). A decline in LVAPD distance is associated with reduced LV function [38]. Defined anatomical landmarks (interventricular septum, LV lateral point, and apical point) were identified in each set of images, and the displacement of these points was recorded from diastole to systole to calculate LVAPD.

Left mid-ventricular peak and global Eulerian circumferential strain (ε_cc_%) were calculated from the short-axis grid tagged images using the Harmonic Phase software (Osman et al. 1999; Virtue; version 5.4, Myocardial Solutions, Morrisville, NC) commonly used and reported in previous DMD studies [3, 39] The myocardial mesh, representing epicardium and endocardium, was manually drawn on top of tags at end diastole or near end diastole. The software automatically generated the myocardial mesh across frames, from diastole to systole, and manual adjustments were made as needed. Maximum displacement of tags as detected by mesh from diastole to systole for each of the six mid ventricular segments gave rise to peak circumferential strain for each segment. Mean peak composite *ℇcc*%, for the LV was defined as the average of the maximum *ℇcc*% (more negative) produced by six segments. Global *ℇcc*% was calculated by averaging *ℇcc*% produced by the six midventricular segments at systole.

To determine if disease progression leads to development of dyssynchrony in the heart, we calculated the circumferential uniformity ratio estimate (CURE) for the mid ventricular segment. CURE is a dyssynchrony index calculated on a scale of 0 to 1 from tagged images [40], with 1 being completely synchronous and 0 being completely dyssynchronous.

Contrast enhancement was not used so there was no attempt made to draw correlations related to myocardial fibrosis.

### Statistical analysis

Statistical analyses were performed using Graph Pad Prism 7 (version 7.0d; GraphPad Software Inc., La Jolla, CA) for cross-sectional comparisons and R v3.5.2 (R Core Team 2018 with readxl and R2Jags packages) for longitudinal analyses [41–43]. For cross sectional comparison, all 59 subjects’ data was used while 47 individuals’ data acquired at UF was used for longitudinal analysis. Independent t-tests with Welch’s correction were used for all cross-sectional group comparisons. For longitudinal data analysis, a Bayesian linear model with first order autoregressive error was considered:

$$y_{i,t} \sim \left\{ {\begin{array}{*{20}l} {{\text{N}}\left( {\mu_{i,t} ,\;\sigma_{y}^{2} } \right),} \hfill & {t = 0} \hfill \\ {{\text{N}}\left( {\mu_{i,t} + \beta \left( {y_{i,t - 1} - \mu_{i,t - 1} } \right),\;\sigma_{y}^{2} } \right),} \hfill & {t \ge 1} \hfill \\ \end{array} } \right.$$

where

$$\begin{aligned} \mu_{i,t} & = \alpha_{t} + \gamma_{{{\text{loa}}}} {\text{loa}}_{i,t} + \gamma_{{{\text{ace}}}} {\text{ace}}_{i,t} + \delta {\text{age}}_{i}^{0} \\ {\text{loa}}_{i,t} & \sim {\text{Bernoulli}}\left( {\eta_{{{\text{loa}}}} } \right),\;{\text{ace}}_{i,t} \sim {\text{Bernoulli}}\left( {\eta_{{{\text{ace}}}} } \right){\text{ independently}}{.} \\ \end{aligned}$$

**Fig. 1:**
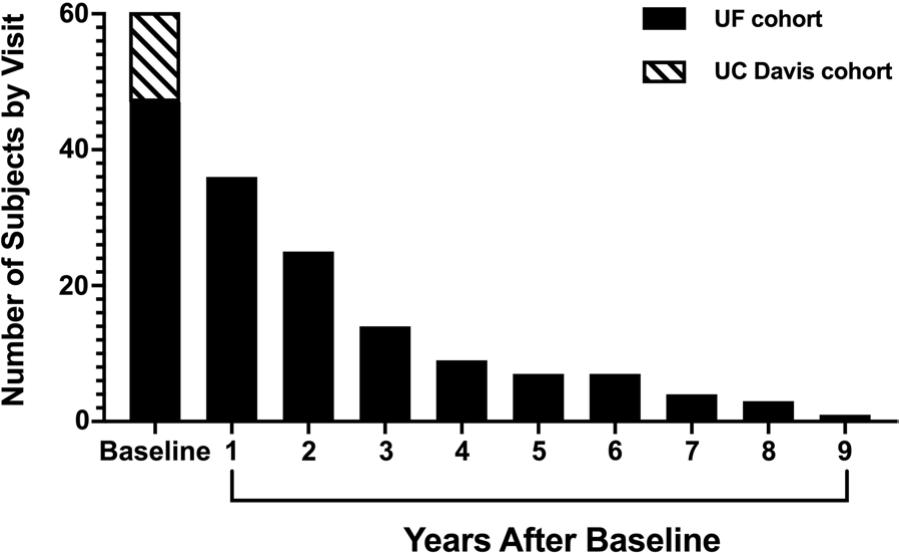
Number of DMD subjects by each timepoint

Here $y_{i,t}$, ${\text{loa}}_{i,t}$ and ${\text{ace}}_{i,t}$ denote the values of a response variable (e.g.,Peak global strain, LVM etc.), indicator of loss of ambulation, and indicator of cardiac drug use, respectively, for the $i$-th individual at time $t$; ${\text{age}}_{i}^{0}$ denotes the baseline age of the $i$ th individual; $\eta_{{{\text{loa}}}}$ and $\eta_{{{\text{ace}}}}$ denote the probability of losing ambulation and probability of administering a cardiac drug respectively; $\delta$, $\gamma_{{{\text{loa}}}}$, $\gamma_{{{\text{ace}}}}$ are the regression parameters associated with baseline age, loss of ambulation and ACE respectively, and $\alpha_{t} - \alpha_{0}$ [$(\alpha_{t} =$ response at time t); ($\alpha_{0} = {\text{response at baseline}}$) measures the average difference between the response at time $t$ and at the baseline. Independent vague proper priors for the model parameters were considered. More specifically, the prior for the parameters β, γ_loa_, γ_ace_, δ, log σ ^2^ and α_t_, t ≥ 0 were taken to be independent, normal (0, 1002) distributions, and the prior for the parameters η_loa_ and η_ace_ were taken to be independent, uniform (0, 1) distributions. Markov chain Monte Carlo (MCMC) samples from the resulting posterior distribution were generated using JAGS [44]. From these posterior samples, the posterior mean and a 95% credible interval (based on quantiles) were computed for each model parameter and for each of the differences αt − α0, t ≥ 1. A parameter or a parameter difference was considered to be significantly different from zero (with 95% probability) if the associated credible interval did not contain zero. A few subjects (n = 8) had data for more than five visits (up to nine visits) (Figs. 1, 4). Some of the follow ups were lost due to disease progression leading to limitation in travel for study visit or due to bad scan quality. For the longitudinal analysis, we used the data from all visits; however, intercept parameters after the fifth visits were not reported, and we focused on the (marginal) posterior distribution of parameters up to the first five visits. Under this analysis, ignorable missingness (missing at random) was assumed to be ignorable.

In this study, we also looked at the minimal clinically important difference (MCID) to determine if the change in cardiac parameters over 1 year are clinically relevant. We used 1/3 standard deviation at baseline as threshold as reported previously [45].

## Results

### Baseline demographics

Forty-seven individuals with DMD and 16 unaffected controls enrolled in the study at UF, and an additional 12 participants with DMD enrolled in the study at UC Davis. In the UF cohort, six participants were non-ambulatory at baseline, while nine subjects in the UC Davis cohort were non-ambulatory. Fifty-nine percent of participants took angiotensin-converting enzyme (ACE) inhibitors or angiotensin receptor blockers (ARBs) at the start of the study (UF n = 28; UC Davis n = 7). Complete baseline demographics are reported in Table 1.

**Table 1:** Baseline demographics of unaffected controls and individuals with DMD

	Controls	DMD (UF)	DMD (UC Davis)
N	16	47	12
Age (years)	12.1 ± 4.1 Range: 6.0 to 18.3	11.2 ± 3.1 Range: 5.3 to 18.1	13.3 ± 2.8 Range: 8.9 to 17.4
Height (cm)	153.7 ± 22.9	129.3 ± 11.2**^++^	149.3 ± 13.5
Body weight (kg)	47.9 ± 22.2	36.7 ± 13.6*^+^	49.1 ± 12.1
ACE inhibitor/ARB	NA	28	7
Steroids	NA	40	12
Non-ambulatory	NA	6	9

Values reported as mean ± SD. One way ANOVA was used to compare three cohorts

*Significantly different from control at *p* < 0.05

**Significantly different from control at *p* < 0.01

^+^Significantly different from UC Davis at *p* < 0.05

^++^Significantly different from UC Davis at *p* < 0.01 Values reported as mean ± SD

### Baseline differences in cardiac function

**Fig. 2:**
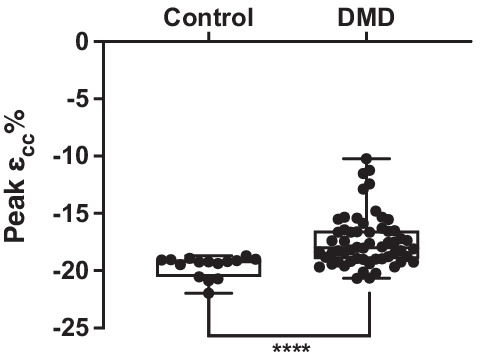
Peak mid ventricular strain (ε_cc_%) in unaffected controls (n = 15) and individuals with DMD (n = 58) at baseline. ****significantly different at *p* < 0.0001. Data is from both UF and UC Davis cohorts

**Fig. 3:**
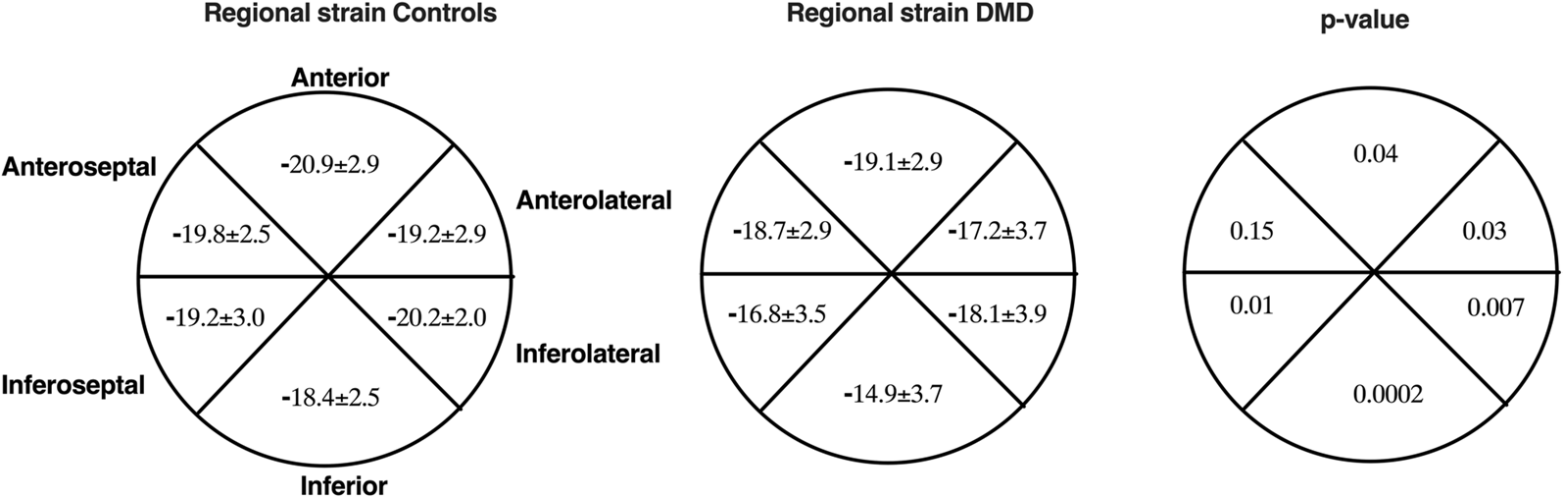
Strain for each LV segment in controls and individuals with DMD (n = 58) at baseline

Peak *ℇcc*% was significantly different in individuals with DMD (− 17.4 ± 2.3%) compared to controls (− 19.6 ± 0.9%; *p* < 0.0001) (Fig. 2) as was global *ℇcc*% (DMD = − 16.6 ± 2.5%; controls = − 19.0 ± 1.1%; *p* < 0.0001) (Additional File 1). When examining strain in each of the six ventricular segments individually, strain was significantly worse in the anterior (*p* < 0.05), inferior septal (*p* < 0.05), inferior (*p* < 0.001), inferior lateral (*p* < 0.01), and anterior lateral (*p* < 0.05) segments (Fig. 3). Circumferential uniformity ratio estimate (CURE) was significantly lower in individuals with DMD (0.93 ± 0.03) compared to controls (0.95 ± 0.01; *p* < 0.001) indicating that the DMD heart is more dyssynchronous (Table 2).

**Table 2:** LV volumetric function and mass in unaffected controls and individuals with DMD at baseline

	Controls (N = 16)	DMD (N = 52)
LVEF (%)	68.3 ± 3.2	63.0 ± 5.3***
LVM	97.9 ± 41.2	79.0 ± 25.5
LVESV	35.6 ± 16.8	28.0 ± 12.1
LVEDV	111.5 ± 46.5	74.7 ± 21.3**
LVMI (gm/m^2^)	73.6 ± 17.5	64.6 ± 11.8
LVESVI (ml/m^2^)	26.1 ± 5.9	23.0 ± 7.2
LVEDVI(ml/m^2^)	82.9 ± 15.7	62.0 ± 12.7***
LVAPD(mm)	− 14.5 ± 1.7	− 11.7 ± 1.5*** (n = 47)
CURE	0.95 ± 0.01	0.93 ± 0.03** (n = 57)

**Significantly different at *p* < 0.01

***Significantly different from controls at *p* < 0.001. Data is from both UF and UC Davis cohorts. Values reported as mean ± SD

Left ventricular ejection fraction (LVEF) at baseline was significantly lower in DMD compared to unaffected controls (*p* < 0.001) (Table 2). LVEDV was lower (*p* < 0.05) in DMD than controls, and there was a trend towards lower LVM (*p* = 0.07) and ESV (*p* = 0.06) (Table 2). After normalization to BSA, LVEDVI remained significantly reduced in DMD (*p* < 0.001). Though these measures were reduced in DMD, all values except for LVEDV and LVEDVI were still in the normal range, as reported in a recent study by van Der Ven et al. [46]. Along with these global cardiac measures of mass and function, we found LVAPD to be significantly reduced in DMD compared to unaffected controls (*p* < 0.0001) (Table 2). Baseline cardiac measures from just the UF cohort are shown in the supplemental data (Additional Files 4, 5 and 6).

To understand the relationship between of loss of ambulation (LOA) and cardiac function, we compared cardiac function between ambulatory (n = 44) and non-ambulatory (n = 15) DMD subjects at baseline. Of the cardiac parameters measured, strain [global (*p* = 0.11) and peak (*p* = 0.15)] were found to show a trend to be deteriorated in non-ambulatory individuals (Table 3) while CURE was significantly deteriorated (*p* < 0.05). LVM was significantly higher in non-ambulatory participants (*p* < 0.01), but this significance was lost when mass was normalized to BSA (Table 3). Also, LVEDVI (*p* < 0.01) and LVESVI (*p* < 0.05) were significantly lower in non-ambulatory individuals (Table 3).

**Table 3:** Comparison of cardiac function in ambulatory to non-ambulatory DMD subjects at baseline

	Ambulatory	Non-ambulatory
Age	10.9 ± 3.1	14.1 ± 1.8
Peak (ε_cc_%)	− 17.9 ± 1.8 (n = 42)	− 16.5 ± 2.8 (n = 15)
LVEF (%)	62.7 ± 4.5( n = 37)	64.1 ± 6.6 (n = 15)
LVM	74.3 ± 28.0 (n = 37)	90.5 ± 12.2** (n = 15)
LVESV	27.6 ± 13.5 (n = 37)	28.9 ± 7.7 (n = 15)
LVEDV	72.6 ± 23.6 (n = 37)	79.8 ± 13.3 (n = 15)
LVMI (gm/m^2^)	65.6 ± 12.2 (n = 37)	62.0 ± 10.6 (n = 15)
LVESVI (ml/m^2^)	24.3 ± 7.2 (n = 37)	19.8 ± 6.2* (n = 15)
LVEDVI (ml/m^2^)	64.9 ± 12.5 (n = 37)	54.5 ± 10.3** (n = 15)
LVAPD (mm)	− 11.7 ± 1.5 (n = 32)	− 11.5 ± 1.2 (n = 15)
CURE	0.94 ± 0.02 (n = 42)	0.91 ± 0.04* (n = 15)

*Significantly different at *p* < 0.05. Data is from both UF and UC Davis cohorts. Values reported as mean ± SD

**Signifcantly different at* p* < 0.01

### Longitudinal changes in cardiac function

**Fig. 4:**
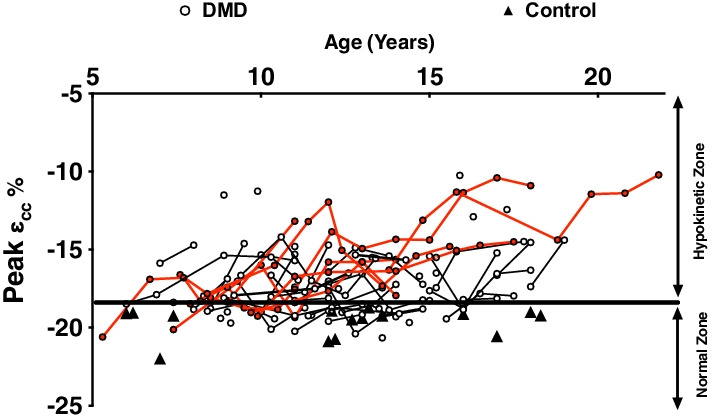
Longitudinal changes in peak strain in DMD. Solid line for peak strain was defined based on 2 SD from mean control value. Red lines indicate subjects with more than 5 years data. Filled triangles are unaffected control values

Both peak (Table 4, Fig. 4) and global *ℇcc* (%) (Additional Files 2 and 3) significantly worsened over the period of 5 years with an absolute change of 3.0% and 2.7%, respectively. Eighty percent of participants with DMD had global strain values considered hypokinetic (Additional Files 2 and 3) (above − 17%; as defined by HARP straining module), and strain showed worsening with age. Further, on examining the six mid-ventricular segments individually, inferior segments showed significant declines over 5 years with absolute changes of 3.2% in the inferoseptal segment, 4.7% in the inferior segment, and 3.8% in the inferolateral segment (Table 4).

**Table 4:** Longitudinal change in composite and regional strain for mid ventricle of DMD

	Baseline	1 year	2 years	3 years	4 years	5 years
Peak (ε_cc_%)	− 17.9 (− 18.4, − 17.4)	− 17.0* (− 17.6, − 16.4)	− 16.7* (− 17.4, − 15.9)	− 17.0 (− 18.0, − 16.0)	− 16.0* (− 17.2, − 14.7)	− 14.9* (− 16.4, − 13.5)
Anterior (ε_cc_%)	− 19.4 (− 20.4, − 18.5)	− 18.7 (− 19.8, − 17.6)	− 19.6 (− 20.9, − 18.2)	− 20.5 (− 22.2, − 18.8)	− 18.6 (− 20.8, − 16.5)	− 16.5* (− 19.1, − 13.9)
Anterolateral (ε_cc_%)	− 17.7 (− 18.6, − 16.9)	− 17.1 (− 18.1, − 16.1)	− 18.5 (− 19.7, − 17.3)	− 17.6 (− 19.2, − 16.1)	− 17.7 (− 19.6, − 15.8)	− 17.6 (− 19.9, − 15.2)
Anteroseptal (ε_cc_%)	− 18.9 (− 19.7, − 18.1)	− 17.8 (− 18.8, − 16.8)	− 17.8 (− 19.0, − 16.5)	− 18.1 (− 19.7, − 16.5)	− 18.4 (− 20.3, − 16.4)	− 16.6 (− 18.9, − 14.2)
Inferior (ε_cc_%)	− 15.4 (− 16.4, − 14.5)	− 14.7* (− 15.9, − 13.5)	− 13.6* (− 14.9, − 12.2)	− 13.3* (− 15.1, − 11.5)	− 12.1* (− 14.5, − 9.8)	− 10.1* (− 12.9, − 7.4)
Inferolateral (ε_cc_%)	− 18.6 (− 19.7, − 17.5)	− 17.1* (− 18.5, − 15.7)	− 15.0* (− 16.7, − 13.3)	− 15.9* (− 18.0, − 13.7)	− 14.9* (− 17.6, − 12.2)	− 14.2* (− 17.5, − 11.0)
Inferoseptal (ε_cc_%)	− 17.1 (− 18.0, − 16.3)	− 16.0 (− 17.1, − 15.0)	− 15.8 (− 17.1, − 14.6)	− 16.4 (− 18.0, − 14.8)	− 14.5* (− 16.5, − 12.4)	− 13.5* (− 16.0, − 11.1)

*Significant effect at 95% level. Values reported as estimates with 95%CI upper and lower limit

When examining longitudinal changes in volumetric functions and mass, there was no significant change in LVESV, LVEDV, and LVM over 5 years (Table 5). LVEDVI showed a significant decline over 4 years after which it showed a trend towards increase from the 4th to 5th year. LVEF dropped significantly over 5 years with decrease in mean values of of 7.9% (Table 5). Along with these findings, we also found an 18% decline in LVAPD (Table 5). Numerical but nonsignificant decreases were seen for LVMI and CURE.

**Table 5:** Longitudinal change in Left Ventricle Volumetric function and Mass of DMD

	Baseline	1 year	2 years	3 years	4 years	5 years
LVAPD (mm)	− 11.8 (− 12.3, − 11.2)	− 10.9* (− 11.6, − 10.2)	− 11.4 (− 12.3, − 10.5)	− 11.0 (− 12.1, − 9.9)	− 11.3 (− 12.7, − 10.0)	− 9.6* (− 11.3, − 7.9)
CURE	0.94 (0.93, − 0.95)	0.92 (0.91, − 0.93)	0.93 (0.92, − 0.95)	0.92 (0.90, − 0.94)	0.92 (0.90, − 0.94)	0.91* (0.88, − 0.93)
LVEF (%)	63.1 (62.1, 64.2)	61.9 (60.4, 63.4)	61.4 (59.5, 63.3)	59.2* (56.8, 61.7)	59.1* (55.7, 62.6)	55.2* (51.4, 58.9)
LVM (gm)	73.5 (69.3, 77.6)	77.7 (71.3, 84.1)	83.7* (75.3, 92.0)	85.0* (73.8, 95.9)	90.4* (75.0, 105.8)	86.6 (69.0, 104.3)
LVMI (gm/m^2^)	64.2 (61.9, 66.6)	65.0 (61.3, 68.6)	66.2 (61.1, 71.3)	62.4 (55.7, 69.1)	62.3 (53.7, 71.0)	58.7 (49.0, 68.3)
LVESV (ml)	26.2 (24.5, 27.9)	27.3 (24.7, 30.0)	29.7* (26.2, 33.1)	28.5 (23.9, 33.0)	26.8 (20.4, 33.2)	33.3 (26.0, 40.5)
LVESVI (ml/m^2^)	23.3 (24.4, 28.0)	23.3 (24.1, 29.7)	24.8 (25.9, 33.5)	22.8 (23.6, 33.6)	18.9 (20.4, 33.5)	23.3 (25.9, 40.9)
LVEDV (ml)	70.6 (67.2, 74.1)	71.1 (65.8, 76.4)	75.9 (68.9, 82.7)	69.0 (60.0, 78.2)	64.5 (51.8, 77.1)	72.5 (57.8, 86.6)
LVEDVI (ml/m^2^)	62.5 (59.5, 65.5)	60.2 (55.7, 64.7)	63.4 (57.1, 69.6)	53.6* (45.3, 61.7)	45.3* (34.9, 55.8)	50.2* (38.4, 61.7)

*Significant effect at 95% level. Values reported as estimates with 95%CI upper and lower limit

### Effect of age, loss of ambulation, and medication on cardiac function

We examined the effect of covariates including baseline age, ambulatory status, and cardioprotective medication use (ACE inhibitor/ARB) on longitudinal changes in cardiac function. Table 6 reports the effect of the presence of each covariate on the average value of each cardiac parameter at a given point in time. Loss of ambulation (LOA) trended towards worsening of peak ε_cc_(%), and year of age at baseline had a significant worsening effect. Cardioprotective medication showed a trend toward a protective improvement in peak ε_cc_(%) and volumetric and mass parameters, and included significant improvements in LVM, ESV and EDV. An increase in age by 1 year was found to be a significant predictor of change in strain, LV mass, ESV and EDV volume. (Table 6).

**Table 6:** Effect of covariates age, loss of ambulation and medication status on cardiac functions in DMD

	LOA	ACE/ARB	Baseline age
Peak (ε_cc_%)	0.2	− 0.6	0.2*
CURE	− 0.02	0.0	0.0
LVEF (%)	0.4	0.9	− 0.3
LVM (gm)	6.5*	− 5.6*	4.8*
LVMI (gm/m^2^)	0.9*	− 2.1	3.7
LVESV (ml)	− 0.7	− 2.6*	1.4*
LVESVI (ml/m^2^)	− 2.9*	− 2.0	0.1
LVEDV (ml)	− 1.1	− 5.9*	3.2*
LVEDVI (ml/m^2^)	− 6.4	− 4.2	− 0.4

*Significant effect at 95% level

## Discussion

The main aim of this study was to examine longitudinal changes in cardiac function in DMD. To our knowledge, this is the first prospective cardiac MR study examining cardiac remodeling in a large cohort of individuals with DMD in a longitudinal fashion over a period of five years or more. We found strain to be a sensitive marker to quantify degree of cardiac pathology and its progression over five years in this patient population when most of the global cardiac functions were still relatively preserved and within normal ranges [46]. Circumferential strain was affected earlier than other cardiac parameters when comparing the DMD population to unaffected controls. Evaluating the effect of different covariates, cardioprotective medications in the form of ACE inhibitors/ARBs were found to have a positive impact on cardiac function, while an increase in age and LOA had a negative impact.

Our study provides valuable, longitudinal natural history data of changes in LV function in individuals with DMD in the context of standard of care clinical management. The data provides a valuable reference base for safety measures and evaluation of therapeutic interventions in a progressive disease using non contrast based imaging methods and will help with design and interpretation of clinical trials for DMD cardiomyopathy. Moreover, this study included young DMD boys prior to development of overt cardiomyopathy, which is critical based on the number of preclinical studies that have shown that early interventions may yield a better outcome [19, 47].

### Strain and volumetric cardiac function decline in DMD

In our study, we found that volumetric cardiac function, strain, CURE, LVAPD, and LV mass were all different in DMD in comparison to the unaffected control group. We found a significant worsening (less negative) of both peak and global LV strain in DMD. We also found a significant difference in LVEF, LVEDV, and LVEDVI compared to controls at baseline. Though the values for these volumetric and mass parameters were lower than the unaffected population, they were still within the normal range except for LVEDV. Our results agree with previous cross sectional studies [27, 28, 32, 48] which have found composite circumferential strain on tagged and CINE images (using feature tracking) strain to be significantly worse in comparison to healthy controls and to be affected earlier in the disease process prior to structural remodeling [28, 49]. These findings emphasize the importance of measuring strain in this patient population at an early stage of the disease. A study by Khan et al. [50] and a previous study by Lee et al. [51] have reported that individuals with DMD develop cardiac atrophy as evidenced by reduced LVMI. Our findings report a similar trend in the decline in LVM and LVMI. However, these measures were not significantly different from that of controls, which could be attributed to the fact that our participants with DMD were relatively younger with better cardiac functions than those reported in the previous studies. Also, we found a significant decrease in LVEDV and LVEDVI in participants with DMD, and these values were out of normal range. We believe that this change in end diastolic volume can be due to smaller hearts and development of diastolic dysfunction. Further studies are required to understand the pathology and cause of reduced LVEDV out of normal range in this patient population.

To understand changes in the long axis of the heart and circumferential mechanical dyssynchrony, we examined LVAPD (a marker of systolic and diastolic dysfunction) and CURE, respectively. Compared to controls, participants with DMD had lower values for LVAPD, indicating reduced AV plane displacement. Different studies have used AV plane displacement as a marker of adverse cardiac events, and it was found to decline with disease progression and age [38, 52]. The decrease in AV displacement has been reported to be due to a reduction in contractility and relaxation of the heart [38, 52], leading to lower diastolic filling pressure and reduced diastolic volume. In our study, we found that both LVEDV and LVEDVI were significantly lower than controls while there was no significant decline in LV mass and LVESV. This can be due to development of stiffness in heart at an early stage, which in turn leads to less relaxation of heart and less filling pressure, resulting in reduced LVEDV.

In order to determine if all regions of the mid LV were affected to the same extent, we examined regional differences in circumferential strain over time in six mid ventricle segments as defined by the American Heart Association [53]. In comparison to controls, 5 out of 6 mid ventricle segments presented with worsened strain (less negative) in DMD. Our results are in agreement with a previous study by Hor et al. [28], where certain segments produced less negative strain in DMD before any evidence of gadolinium enhancement. The inferior lateral free wall was affected more and earlier than others, which is consistent with autopsy studies describing more fibrotic accumulation in the inferior wall segment [34, 54]. These findings emphasize the importance of measuring composite and regional strain in this patient population and indicate that despite normal LV volumetric function, pathological cardiac remodeling begins at an early age in this patient population supporting cardiac strain as an early biomarker for cardiac dysfunction in DMD.

### Longitudinal changes in cardiac function

In this study we examined changes in cardiac function longitudinally for up to 9 years with statistical analysis over 5 years. To our knowledge, this is the first prospective study examining the changes in cardiac function/remodeling in DMD longitudinally over a period greater than 3 years. In our cohort, we found a consistent decrement in both global and peak composite strain over the 5 years. Along with composite strain, regional strain for inferior segments also showed a significant decline over this period. These findings emphasize that inferior free wall segments of the heart, which had significantly worse strain production in comparison to controls at baseline, are more affected as the disease progresses. This worsening of strain can be due to combination of damage in myocardium leading to cardiac remodeling due to disease pathology [3] and changes in heart hemodynamics which, in turn, can further worsen the disease pathology [55, 56].

Along with strain, we also observed a significant decline in EF over 5 years. The decrease in LVEF can be attributed to various factors, including a loss in heart contractility, dilation of the ventricular cavity, or lower pumping capacity of the heart due to a reduction in LVEDV. We believe this decline in LVEF is dependent on two factors. First, in the early stage, because of a decrease in contractility, there is the development of both systolic and diastolic dysfunction leading to lower LVEDVI (significant decline over 4 years). Second, in the later stage, along with previous abnormal changes, there is the development of dilated cardiomyopathy leading to an increase in LVEDV and, in turn, reduction in LVEF. Of note, although LVEF and other volumetric function show worsening over time, the values still fell within the normal range. On the other hand, strain values (both global and composite) were well below normal at baseline and declined over the 5 years.

This study also looked at the minimal clinically important difference (MCID) on all the cardiac parameters over 1 year. MCID helps us identify whether the deterioration in an outcome measure is clinically relevant for this population. Different studies have reported various methods to calculate MCID using standard error, standard deviation, or anchor-based methods. In this study, we used 1/3 SD at baseline as a threshold [45], and we found that 8 out of 17 cardiac parameters examined showed clinical relevance, including peak strain, global strain, LVAPD, CURE, regional segmental strain (anterior septal strain, inferior septal strain, inferior strain), and ejection fraction. But, when we used ½ SD as a threshold reported previously [57], we found only three parameters CURE, peak strain, and global strain showing the clinically relevant change. Based on these findings, other than strain, CURE was the only parameter which was found to be clinically meaningful at both the thresholds, indicating that this can be a promising tool to understand cardiac pathology along with strain in this patient population. Future studies need be performed to determine its clinical utility in larger cohorts spanning a wider age range.

### Heterogeneity in longitudinal disease progression in DMD and the effect of covariates

One of the critical findings of this study was that we found significant heterogeneity among the subjects’ strain values (Fig. 4), with some subjects showing decrements in strain at an early age (less than 7 years of age) and others showing maintenance/improved strain (more negative) even at an older age. Various factors may contribute to this presentation such as presence of genetic modifiers [58, 59], type of mutation in the dystrophin gene [60], skeletal muscle involvement [61], and timing and role of cardioprotective medication [62, 63]. To understand some of these possible contributions, we examined the effect of loss of ambulation, age, and cardioprotective medication as covariates. When comparing ambulatory and non-ambulatory participants both cross sectionally and longitudinally, we found a trend towards worsening of strain values and significant changes in LV mass and volume which was associated with LOA. These findings indicate that non ambulatory subjects present with worse cardiac function and cardiac atrophy as is evident by decreases in LVMI, LVESVI and LVEDVI. Previously, a study conducted by Posner et al. [61] found a significant relationship between skeletal muscle function and cardiac output measures, but there was no cause and effect relationship. One of the confounding issues when comparing ambulatory and non-ambulatory participants across different centers can be ascribed to the difference in definition of LOA [58, 64, 65]. Indeed, if we use an alternative definition of LOA (unable to complete 10 m run/walk under 10 s) as initially defined in UC Davis Cohort (NCT02964377; 11/16/2016) three of the ambulatory participants with the worst cardiac function would be reassigned to the LOA group resulting in significant changes in all cardiac parameters with LOA. This discrepancy emphasizes the need to develop a consistent universal definition of LOA so that data can be interpreted and analyzed across different trials and studies.

In the relatively young cohort of our study, we also found a positive trend for the impact of ACE inhibitors/ARBs on cardiac function. In a study by Raman et al. [33], they also found that a combination of ACE inhibitors/ARBs and eplerenone attenuated decline in cardiac function in DMD. The ACE inhibitor/ARB with eplerenone group reported 1% change in peak strain which was found to be clinically significant; however, this effect might be short-lived as disease pathology eventually negates the positive cardiac remodeling caused by these drugs. A study by Wang et al. [66] and Packer [67] have reported similar findings where they found the effect of ACE inhibitors/ARBs to be transient in a DMD cohort and an adult heart pathology population.

## Limitations

There were certain limitations associated with the study. The goal of the study was not to draw correlations to fibrosis, and as such, contrast was not used and no quantitative measurement of native T_1_ and T_2_ were obtained in this study. These measurements, in combination with diffusion, could be used to help interpret the strain findings in the context of myocellular and extramyocellular remodeling of the myocardium. It will be important for future studies utilizing quantitative contrast enhancement techniques to delineate the changes in extracellular volume, native T_1_, T_2_ , and their relationship to strain. The study did not acquire information about cardiac hemodynamics (including blood pressure) or ECG changes which could potentially help better understanding the relationship between change in strain and volumetric function with disease progression. Also, this study did not acquire any strain data for basal and apical segments which can help interpret changes in rotational mechanics of the heart (68). In this study, we examined the longitudinal effect of covariates including age, LOA, and cardioprotective medications. Unfortunately, we could not compare the trajectories of age-matched patients with cardioprotective medication to patients who are not receiving any drugs and ambulatory to non-ambulatory status because of the limited sample size. While we examined the effect of cardioprotective medication as a covariate on cardiac functions and structure, we were not able to determine the effect of changes in type of medication or drug dosage.

## Conclusion

These findings emphasize the importance of circumferential strain as a sensitive measure to monitor disease progression in DMD as it was able to detect cardiac pathology at an early age with children as young as 5 years of age. We observed that decrements in strain occurred while other measures, such as ejection fraction, showed a decline but were still within the normal range. Decline in strain at both composite and regional levels over 1 year showed minimally clinically important differences with disease progression. The findings also highlight the heterogeneity in cardiac progression in this patient population, which is especially important in designing a clinical trial and understanding the response to therapeutic intervention. Further, these results reveal that cardiac involvement in DMD starts at a relatively early age, and support that the notion that cardiac evaluation and therapeutic intervention should start early.

## Supplementary Information

**Additional file 1:** Global mid ventricular strain (εcc %) in unaffected controls (n=15) and individuals with DMD (n=58) at baseline.

**Additional file 2:** Longitudinal changes in global strain in DMD. Solid line for global strain was defined based on normal zone cut off of -17% as given by HARP software. Red lines indicates subjects with more than 5 years data, filled triangles represent unaffected controls.

**Additional file 3:** Longitudinal change in global strain for mid ventricle of DMD.

**Additional file 4:** Peak and global mid ventricular strain (εcc %) in unaffected controls (n=15) and individuals with DMD (n=46) at baseline (UF Cohort).

**Additional file 5:** Strain for each LV segment in controls and individuals with DMD (n=46) at baseline (UF cohort).

**Additional file 6:** Comparison of cardiac function in control and DMD subjects at baseline (UF Cohort).

## Data Availability

The datasets used and/or analyzed for this manuscript are available from the corresponding author (Dr. Glenn Walter) on reasonable request.
