# Supplementary material for: Longitudinal changes in cardiac function in Duchenne muscular dystrophy population as measured by magnetic resonance imaging
Source: BMC Cardiovasc Disord. 2022 Jun 9;22:260. doi: 10.1186/s12872-022-02688-5 (PMC9185987; doi:10.1186/s12872-022-02688-5)
Supplement: Supplementary file 1 — Additional file 1: Global mid ventricular strain (εcc %) in unaffected controls (n=15) and individuals with DMD (n=58) at baseline. [file 12872_2022_2688_MOESM1_ESM.docx]

Additional File 1: Global mid ventricular strain (ε_cc_ %) in unaffected controls **(n=15)** and individuals with DMD **(n=58)** at baseline

**** significantly different at p<0.0001
