# Supplementary material for: Longitudinal changes in cardiac function in Duchenne muscular dystrophy population as measured by magnetic resonance imaging
Source: BMC Cardiovasc Disord. 2022 Jun 9;22:260. doi: 10.1186/s12872-022-02688-5 (PMC9185987; doi:10.1186/s12872-022-02688-5)
Supplement: Supplementary file 2 — Additional file 2: Longitudinal changes in global strain in DMD. Solid line for global strain was defined based on normal zone cut off of -17% as given by HARP software. Red lines indicates subjects with more than 5 years data, filled triangles represent unaffected controls. [file 12872_2022_2688_MOESM2_ESM.docx]

Additional File 2: Longitudinal changes in global strain in DMD. Solid line for global strain was defined based on normal zone cut off of -17% as given by HARP software. Red lines indicates subjects with more than 5 years data, filled triangles represent unaffected controls.
