# Supplementary material for: Longitudinal changes in cardiac function in Duchenne muscular dystrophy population as measured by magnetic resonance imaging
Source: BMC Cardiovasc Disord. 2022 Jun 9;22:260. doi: 10.1186/s12872-022-02688-5 (PMC9185987; doi:10.1186/s12872-022-02688-5)
Supplement: Supplementary file 3 — Additional file 3: Longitudinal change in global strain for mid ventricle of DMD. [file 12872_2022_2688_MOESM3_ESM.docx]

Additional File 3: Longitudinal change in global strain for mid ventricle of DMD

|  | **Baseline** | **One year** | **Two Years** | **Three Years** | **Four Years** | **Five Years** |
| --- | --- | --- | --- | --- | --- | --- |
| **Global (ε_cc_ %)** | -17.1  (-17.6,-16.6) | -15.9*  (-16.6,-15.2) | **-16.0***  (-16.8,-15.1) | -16.0  (-17.1,-14.9) | **-15.2***  (-16.6,-13.9) | **-14.4***  (-16.0,-12.7) |
