# Supplementary material for: Longitudinal changes in cardiac function in Duchenne muscular dystrophy population as measured by magnetic resonance imaging
Source: BMC Cardiovasc Disord. 2022 Jun 9;22:260. doi: 10.1186/s12872-022-02688-5 (PMC9185987; doi:10.1186/s12872-022-02688-5)
Supplement: Supplementary file 4 — Additional file 4: Peak and global mid ventricular strain (εcc %) in unaffected controls (n=15) and individuals with DMD (n=46) at baseline (UF Cohort). [file 12872_2022_2688_MOESM4_ESM.docx]

Additional File 4: Peak and global mid ventricular strain (ε_cc_ %) in unaffected controls (n=15) and individuals with DMD (n=46) at baseline (UF Cohort)

**** significantly different at p<0.0001
