# Supplementary material for: Longitudinal changes in cardiac function in Duchenne muscular dystrophy population as measured by magnetic resonance imaging
Source: BMC Cardiovasc Disord. 2022 Jun 9;22:260. doi: 10.1186/s12872-022-02688-5 (PMC9185987; doi:10.1186/s12872-022-02688-5)
Supplement: Supplementary file 5 — Additional file 5: Strain for each LV segment in controls and individuals with DMD (n=46) at baseline (UF cohort). [file 12872_2022_2688_MOESM5_ESM.docx]

Additional File 5: Strain for each LV segment in controls and individuals with DMD (n=46) at baseline (UF cohort)

* significantly different at p<0.05, *** significantly different at p<0.001
