# Supplementary material for: Longitudinal changes in cardiac function in Duchenne muscular dystrophy population as measured by magnetic resonance imaging
Source: BMC Cardiovasc Disord. 2022 Jun 9;22:260. doi: 10.1186/s12872-022-02688-5 (PMC9185987; doi:10.1186/s12872-022-02688-5)
Supplement: Supplementary file 6 — Additional file 6: Comparison of cardiac function in control and DMD subjects at baseline (UF Cohort). [file 12872_2022_2688_MOESM6_ESM.docx]

Additional File 6: Comparison of cardiac function in control and DMD subjects at baseline (UF Cohort)

|  | **Controls (N=16)** | **DMD (N=41)** |
| --- | --- | --- |
| **EF (%)** | **68.3± 3.1** | **63.0± 4.3***** |
| **LVM** | **97.9± 41.2** | **73.9±19.5*** |
| **ESV** | **35.6±16.8** | **26.4±7.4** |
| **EDV** | **111.5± 46.5** | **70.9±15.3**** |
| **LVMI (gm/m^2^)** | **73.6±17.5** | **64.1±9.8** |
| **ESVI (ml/m^2^)** | **26.1±5.9** | **23.1±5.6** |
| **EDVI(ml/m^2^)** | **82.9±15.7** | **62.5±11.7**** |
| **LVAPD(mm)** | **-14.5±1.8** | **-11.7±1.5***(n=36)** |
| **CURE** | **0.95±0.01** | **0.93±0.02*(n=46)** |

* significantly different at p<0.05, ** significantly different at p<0.01, *** significantly different at p<0.001
